# Post-traumatic stress disorder and resilience among adult burn patients in Pakistan: a cross-sectional study

**DOI:** 10.1186/s41038-018-0110-7

**Published:** 2018-02-12

**Authors:** Akhtar Bibi, Sundas Kalim, Muhammad Adnan Khalid

**Affiliations:** 1 Department of Psychology, Mental Health Research and Treatment Center, Ruhr University , Bochum, Germany; 2 National University of Modern Language, Islamabad, Pakistan; 3 Institute for Sport Sciences and Psychology, Westfälische Wilhelms-Universitat , Munster, Germany

**Keywords:** Adult, Burn injury, Gender, Post-traumatic stress disorder, Resilience

## Abstract

**Background:**

Post-traumatic stress disorder (PTSD) is one of the major psychological disorders developed after burn injuries, though this subject of burn injuries and their destructive chronic psychological impact are not considered as thoughtfully in developing countries like Pakistan. Hence, the current study investigated the relationship between PTSD symptoms and resilience among burn patients in Pakistan, exploring the variance occurrence of the two variables concerning male burn patients and female burn patients.

**Methods:**

Seventy burn patients from three burn units of Rawalpindi and Islamabad, Pakistan, during January 2015 to September 2015 were recruited. Patients with psychiatric disorder which would restrict the study procedures were excluded from the study. PTSD symptoms of burn patients were measured by PTSD CheckList-Civilian Version (PCL-C) and resilience was measured by Connor-Davidson Resilience scale (CD-RISC). Spearman’s Rank-Order correlation was used to analyze the relationship between symptoms of PTSD and resilience in burn patients, and analysis of covariance (ANCOVA) was applied to analyze the gender difference in symptoms of PTSD and level of resilience.

**Results:**

Negative correlation between PTSD and resilience among burn patients was found (*r* = − 0.72, *p* < 0.001). Moreover, significant gender differences were observed on PTSD symptoms and resilience between male and female burn patients when demographic variables such as age, socioeconomic status, marital status, and educational background were controlled. Female burn patients showed more PTSD symptoms (*η*^2^ = 0.18, *p* < 0.001) and less resilience (*η*^2^ = 0.25, *p* < 0.001) when compared to male burn patients.

**Conclusions:**

PTSD and resilience were negatively correlated in burn patients. Female burn patients have more PTSD symptoms and lower resilience compared to male burn patients.

## Background

Severe physical trauma not only is a threat to individual’s safety but also causes physical and psychological health hazards. Burn injuries are generally associated with terrible life events which are mostly accidental and extremely stressful.

Burn injuries influence individual’s ability to cope with stresses of life and interact with the outer world. Most of the burn patients develop acute stress disorder and post-traumatic stress disorder (PTSD). Many burn victims also experience problems in accepting their new appearance and are unable to perform their social and occupational activities [1]. They start perceiving themselves from the perspective of others, which is a painful experience for them. In addition, patient with burn injuries may also lose their earning sources while facing the financial pressures of payment for medical surgery and other procedures involved for their treatment [2]. It has been reported that some burn patients re-experienced the burning trauma and developed repetitive thoughts within one month. They also experienced symptoms of hyper arousal, avoidance, and emotional problems which lead to stress and impaired functioning [3].

Moi et al. [4] conducted open, in-depth interview of 20 burn patients in their qualitative study in the national burn center in Norway. They found that burn patients avoided their first reflection of body after the removal of dressing. They were shocked at their unfamiliar body image. Their skin was fragile, and they could not feel warmth and coldness. In addition to physical health issues, they also developed psychological distresses. They discussed how they experienced feeling of social withdrawal, isolation, and stigmatization [4]. Other studies showed that 19% of burn patients developed acute stress disorder whereas 36% of the patients developed PTSD after burn injury, which is characterized by the re-experience of the trauma events, avoidance of trauma-related stimuli, increased negative thoughts or feelings, and hyper arousal [5]. It usually occurs 3 to 6 months after a burn injury or even later and continues for years, and has long-lasting effects on the well-being and quality of patients’ lives [6]. Destructive nature of burn injuries requires a long-term rehabilitation and patience [7].

Though PTSD is one of the major psychological disorders developed after burn injuries, very few studies have been conducted to address this important psychological disorder in developing countries like Pakistan. In Pakistan, stove burn, accidental burns, domestic violence, and acid throwing attacks have increased in recent years [8]. The main reasons of burn injuries are typically domestic issues and accidents. According to Farooq et al., missmanagement of kerosene pressure stoves, lack of education, obliviousness to harmless practice techniques, and poor socioeconomic status are associated with high occurrence of accidental burns in Rawalpindi, Pakistan [9]. The Progressive Association reported 8000 cases of women who were burnt by acid assaults, kerosene and stoves since 1994 in Islamabad and a 200-mile radius [10].

Positive mechanisms like resilience are positively linked to enhanced mental health [11]. It is the capacity of the individual to cope with the difficulties of life and maintain positive functioning [12]. It includes sound adaptation and individual development in varying and challenging situations. Resilient people are assertive, have good social skills, and better self-control over their emotions [13]. People who are less resilient cannot cope with traumatic situations and develop depression, anxiety, substance abuse and PTSD [14]. Resilience can be produced, sustained or reduced [15]. Due to these features, resilience decreases the damaging effects of PTSD. Several recent studies [16, 17] have revealed that resilience mediate the association between predictive causes and symptoms of PTSD among several populations.

In addition, gender may play a role in the level of PTSD symptoms and resilience. It has been found that female participants develop symptoms of PTSD more frequently than male participants [18]. Research in developing countries such as India found that women aged 16–35 years experience burn accidents more than men [19]. In Pakistan, female burn patients are also likely to experience more burn incidents and psychological distress [8, 9]. Furthermore, some other studies have also revealed that after the trauma, women were at higher risk of receiving a diagnosis of PTSD than men [20] and have lower resilience level than male burn patients [21].

This subject of burn injuries and their destructive chronic psychological impact are not considered thoughtfully in Pakistan. Hence, the current study addresses these issues and emphasizes on assessment of symptoms of PTSD and significance of resilience across both genders. It also can be useful for mental health professionals and clinicians in dealing with burn patients to reduce their symptoms of PTSD and enhance their resilience.

Resilience plays an important role in dealing with stressful situations and reduces detrimental effects of mental health challenges. In the cultural context of Pakistan, women have limited resources to deal with challenges of life and, as a result, experience more psychological disorders. In the light of cultural background, it was hypothesized that there would be a negative correlation between PTSD symptoms and resilience among burn patients, female burn patients would score higher on PTSD scale when compared to male patients, and male burn patients would have higher resilience levels when compared to female burn patients.

## Methods

### Participants

Seventy burn patients aged 18-50 years with 10-30% total burn surface area (TBSA) burned from burn units of Rawalpindi and Islamabad, Pakistan, during January 2015 to September 2015 were recruited.

**Fig. 1:**
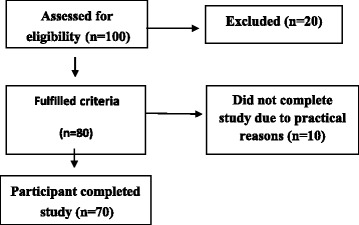
Flow chart of the participants’ recruitment

Patients with psychiatric disorder which would restrict the study procedures were excluded from the study. Totally 100 patients were approached, and 80 of them met the criteria of research, but 10 participants refused to participate due to pain and other practical reasons (preoccupation with treatment complications). Thus, 70 participants willingly participated in the study. It was not possible to improve sample of the study due to limited resources (Fig. 1).

**Table 1:** Frequencies, percentages, median, and interquartile range of demographic variables of patients with burns (*N* = 70)

Variable	Frequency (n)	Percentage (%)	Median	IQR
Age (years)			27.50	13
18–29	39	55.7		
30–40	22	31.4		
41–50	9	12.9		
Gender				
Male	34	48.6		
Female	36	51.4		
Education				
Uneducated	30	42.9		
Metric	26	37.1		
Intermediate	5	7.1		
Graduate	7	10.0		
Post-graduate	2	2.9		
Monthly income (PKR)				
10,000–25,000	34	48.6		
25,000–50,000	19	27.1		
Above 50,000	17	24.3		
Family structure				
Nuclear	39	55.7		
Joint	31	44.3		

*IQR* interquartile range, *PKR* Pakistan Rupees

**Table 2:** Frequencies and percentages of injury and hospitalization details of patients with burns (*N* = 70)

Variable	Frequency (n)	Percentage (%)
Cause of burn		
Gas explosion	35	50.0
Acid burn	5	7.1
Chemical burn	14	20.0
Liquids and steam	11	15.7
Electric burn	5	7.1
Total burn surface area		
10%	10	14.3
10–20%	21	30.0
20–30%	39	55.7
Duration of hospitalization		
Less than 1 month	10	14.3
1 month	31	44.3
2 months	26	37.1
3 months	3	4.3
Surgical intervention		
Debridement	48	68.6
Grafting	22	31.4

### Questionnaire

#### PTSD CheckList-Civilian Version (PCL-C)

PTSD symptoms were accessed by the Urdu version of PCL-C, which was a 17-item scale. PCL-C [22] measured symptoms of PTSD with a 5-point Likert scale ranging from 1 (not at all) to 5 (extremely). Cronbach’s alpha reliability coefficient of the PCL-C was 0.91.

#### Connor-Davidson Resilience Scale (CD-RISC)

Resilience was accessed by the Urdu version of CD-RISC. It is a reliable instrument in measuring resilience among burn patients. The 10-item CD-RISC [23] measured the resilience with a 5-point scale ranged from 0 (not true at all) to 4 (nearly true all of the time). Cronbach’s alpha reliability of CD-RISC was 0.95.

### Procedure

The current study was approved by the ethical committee of the National University of Modern Languages (NUML), Islamabad, Pakistan. A cross-sectional study design was employed. Participants were informed about the purpose of research, voluntariness, and anonymity information and informed consent before filling questionnaires. PCL-C, CD-RISC, and demographic information were collected. The entire survey took approximately 15 to 20 min.

### Statistical analysis

The SPSS (Statistical Package for Social Sciences) 23 software was used to analyze the data. Frequencies and descriptive statistics were tabulated for demographic variables. Spearman’s Rank-Order correlation was used to analyze the association between symptoms of PTSD and resilience in burn patients. Analysis of covariance (ANCOVA) was used to analyze the gender difference in symptoms of PTSD and level of resilience when other demographic variables including age, sociodemographic status, marital status, and educational background controlled.

## Results

### Demographic characteristics of the patients

Missing data (less than 2% of entire data) was replaced by the median value of that item. Frequencies, percentages, median, and interquartile range of demographic variables of patients with burns were presented in Table 1. The major causes of burn injury in women including fire, gas explosion, and acid burn whereas chemical, liquid, and steam burn was more common in male burn patients. Frequencies and percentages of injury and hospitalization details of patients with burns were presented in Table 2.

### Correlation between PTSD and resilience among burn patients

Correlation between PTSD and resilience among burn patients was investigated. Results indicated a strong negative correlation between PTSD and resilience among burn patients (*r* = −0.72, *p <* 0.001)*.*

### Gender difference in PTSD and resilience among burn patients

**Fig. 2:**
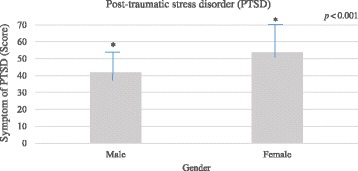
Post-traumatic stress disorder (PTSD) among male and female burn patients. Analysis of covariance (ANCOVA) revealed that gender difference in PTSD among burn patients was statistically significant.

ANCOVA was applied to examine whether the male and female burn patients differed on PTSD and resilience, when other demographics variables including age, sociodemographic status, marital status, and educational background controlled. A significant effect of gender was found on PTSD among burn patients *F* (1, 64) = 14.22, *p <* 0.001, (*η*^2^ = 0.18) indicating that female patients suffered more severe PTSD symptoms than male did (Fig. 2).

**Fig. 3:**
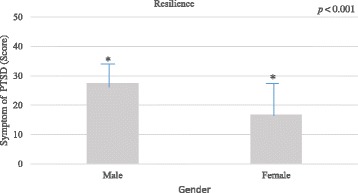
Resilience among male and female burn patients. Analysis of covariance (ANCOVA) revealed that gender difference in resilience among burn patients was statistically significant. *PTSD* Post-traumatic stress disorder

Similarly, there was also a significant effect of gender on resilience among burn patients, *F* (1, 64) = 22.03, *p <* 0.001 (*η*^2^ = 0.25), suggesting that female had generally lower resilience than male (Fig. 3).

## Discussion

The results of the current study showed that symptoms of PTSD and resilience are interrelated in burn patients. Low level of resilience is associated with higher symptoms of PTSD. Gender also significantly contributed in level of PTSD and resilience in burn patients. Burn injury is one of the worst types of injury that one can experience which causes different physical and mental concerns. Moreover, it has been reported that burn patients are more vulnerable to develop psychological problems due to traumatic nature of severe burn injuries [24]. In the present study, a negative correlation among PTSD and resilience was observed in a group of burn patients in Pakistan. It is in line with the previous finding [16, 17] that resilience decrease the likelihood of PTSD. Resilience also enhances the individual’s ability to deal with adverse traumatic situation effectively and defend against PTSD [25].

Kornhaber et al. [7] reviewed resilience in the context of burn injury and rehabilitation, and they found that three main themes are related with the impact of resilience: interpersonal strengths, optimistic coping, and confrontation to trauma symptoms. These themes propose that resilience significantly contribute in the rehabilitation of burn patients throughout their life. Moreover, resilience has been found to play a moderator role on the positive relationship between experiences of violence and re-experiencing symptoms of PTSD [26]. Resilience is positively related with social support; however, burn patients receive low social support from family, associates, and significant others in Pakistan [27]. Therefore, it is significant to include families for the care of burn patients rather than only considering their wounds. Hence, improving their level of resilience or the supports they received could help coping with the stressful events and may lower the symptoms of PTSD.^.^

In the current study, female burn patients scored higher on PTSD compared to male burn patients. The results are consistent with some previous studies. For example, meta-analyses of studies of PTSD showed that in general female participants meet the criteria for PTSD more frequently when compared to males [18]. A possible explanation of this gender differences is that in Pakistan women are not encouraged to express their feelings compared to men (Fig. 3). As a result, they develop psychological disorders more frequently compared to men. Other possible explanation could be lack of supports and bad environment. For instance, in Pakistan, hundreds of women die as a result of stove-burn and burn attacks by their own family members such as husband and in-laws every year. The intention behind this stove burn is to dispose the women and marry again for more economic gain in the form of dowries [28]. Therefore, the background behind the injuries can also impact their mental well-being. In fact, studies have shown that burn patients with serious burn injuries and little social support experienced more symptoms of PTSD [29].

Also, we observed that male burn patients scored higher on resilience as compared to female burn patients. The results in these populations are with culturally significant context. The finding was in line with the research of Banano et al. [21] after the September 11, 2001, extremist attack; they found in a phone survey of adult population (*N* = 2752) in New York City that in stressful situation, men had higher tendency of resilience when compared to women. Men and women vary in their coping style; men have more active coping style compared to women [30]. It is thought that male patients with higher psychological resilience can adjust themselves while keeping accordance with environmental changes, but female burn patients showed avoidance to adjust in new environment [31]. The resilience of burn patients depends upon different factors, such as gender, nature of work, marital status, education, duration, and nature of burn [32]. Expected cultural, social, and gender role makes the women less resistant to traumatic situations and causes different psychological disorders. In Pakistan, women experience burn injuries as a result of domestic violence and interpersonal trauma. Women’s resiliency decreases when they sense that their partner and family are committers. In some way, society and culture also allow this act of brutality. The concept of family and society, as well as traditional, political, and law frameworks like advocacy, becomes substantial to prevention and management. There are possibilities for active coping if one is continually persecuted. Policies should be developed in Pakistan to improve the position of women as valuable members of the society.

There are some limitations of the present study. Firstly, due to the relatively small sample size and the specific participant characters, the results of the current study should be interpreted cautiously when generating to other non-Islamic countries. More studies in different culture groups are encouraged to elucidate the relationship between PTSD and resilience. It appears realistic to be careful in interpreting the results to be limited to the cultural context in which they are produced. Secondly, outcome measure (e.g., CD-RISC) is not generally applied in burn patients and may have deficient sensitivity when used to burn patients. Thirdly, it was a cross-sectional study, and we do not know whether there exists a causal relationship between PTSD and resilience. We suggest that in the future, longitudinal studies should be conducted in the cultural context of Pakistan to investigate whether resilience is a protective factor of PTSD.

Keeping in view the culture of Pakistan, this study would help mental health professionals, policy makers, and social workers in making different strategies for burn patients to address their mental health demands, resilience level, and social support network. Family and peer support play a vital role in the recovery and rehabilitation of burn-injured patients. Appropriate psychological care and social support system may improve the complete clinical effects of burn patients. Culture-based rehabilitation strategies should be planned to improve their resilience level and holistic care. Improving optimism and faith could also help in dealing with trauma and rehabilitation of these patients.

## Conclusions

Current study found that higher symptoms of PTSD are associated with lower resilience. Gender difference in PTSD and resilience among burn patients were observed. Female burn patients have more symptoms of PTSD and lower resilience compared to male. Resilience may be a protective factor of PTSD.
